# Clinical Efficacy of Simulated Vitreoretinal Surgery to Prepare Surgeons for the Upcoming Intervention in the Operating Room

**DOI:** 10.1371/journal.pone.0150690

**Published:** 2016-03-10

**Authors:** Svenja Deuchler, Clemens Wagner, Pankaj Singh, Michael Müller, Rami Al-Dwairi, Rachid Benjilali, Markus Schill, Hanns Ackermann, Dimitra Bon, Thomas Kohnen, Benjamin Schoene, Michael Koss, Frank Koch

**Affiliations:** 1 Vitreoretinal Unit, University Eye Hospital, Frankfurt/Main, Hessen, Germany; 2 VRmagic, Mannheim, Baden-Württemberg, Germany; 3 Institute of Biostatistics and Mathematical Modelling, University Hospital, Frankfurt/Main, Hessen, Germany; 4 University Eye Hospital, Frankfurt/Main, Hessen, Germany; 5 King Abdullah University Hospital, Irbid, Jordan; 6 University Eye Hospital, Heidelberg, Baden-Württemberg, Germany; University of Florence, ITALY

## Abstract

**Purpose:**

To evaluate the efficacy of the virtual reality training simulator Eyesi to prepare surgeons for performing pars plana vitrectomies and its potential to predict the surgeons’ performance.

**Methods:**

In a preparation phase, four participating vitreoretinal surgeons performed repeated simulator training with predefined tasks. If a surgeon was assigned to perform a vitrectomy for the management of complex retinal detachment after a surgical break of at least 60 hours it was randomly decided whether a warmup training on the simulator was required (n = 9) or not (n = 12). Performance at the simulator was measured using the built-in scoring metrics. The surgical performance was determined by two blinded observers who analyzed the video-recorded interventions. One of them repeated the analysis to check for intra-observer consistency. The surgical performance of the interventions with and without simulator training was compared. In addition, for the surgeries with simulator training, the simulator performance was compared to the performance in the operating room.

**Results:**

Comparing each surgeon’s performance with and without warmup trainingshowed a significant effect of warmup training onto the final outcome in the operating room. For the surgeries that were preceeded by the warmup procedure, the performance at the simulator was compared with the operating room performance. We found that there is a significant relation. The governing factor of low scores in the simulator were iatrogenic retinal holes, bleedings and lens damage. Surgeons who caused minor damage in the simulation also performed well in the operating room.

**Conclusions:**

Despite the large variation of conditions, the effect of a warmup training as well as a relation between the performance at the simulator and in the operating room was found with statistical significance. Simulator training is able to serve as a warmup to increase the average performance.

## Introduction

Virtual reality education and training are established tools for building up expertise in many specialities worldwide, such as aviation or medicine.

Khalifa et al [1] found that ophthalmology training programs were struggling to find viable methods of assessing and documenting the surgical skills of trainees, and that the role of virtual reality education and training in future curricula was still uncertain in 2006.

Vitreoretinal surgery training with the ophthalmic surgical simulator Eyesi was introduced in June 2003 when two Eyesi simulators were used for the first time in a teaching lab, during the 5^th^ International Vitreoretinal Symposium (VRS) in Frankfurt. The success of this lab led to the introduction of the dry lab concept: simulation in combination with practice eyes, table microscopes and real surgical machines.

Simulator-based training in the anterior as well as the posterior segment of the eye found its way into training and education curricula rapidly [2].

In particular the anterior segment has been well evaluated in numerous studies [3–9] focusing both on construct validity (in the anterior [10–13] and posterior segment [14]) as well as on the transfer of skills from virtual reality to wet lab training and to the operating room. Feudner et al [15] investigated how capsulorhexis training on Eyesi improved wetlab capsulorhexis performance of surgical novices, while McCannel et al [16] took a step forward by comparing the capsulorhexis performance of residents in the operating room before and after the introduction of simulator-based training and found a significant reduction in complications.

The assessment of surgical performance under special conditions such as sleep deprivation [17–18] or beta-blockade [19] has also been discussed. Gill et al [20] investigated the ability of simulation to predict the actual surgical proficiency of residents and attending physicians for anterior segment surgery.

For the posterior segment, there are no known skills-transfer trials from virtual reality environments to the operation room.Our study evaluates in a prospective setup whether preparation at the simulator affects the surgical performance and whether it allows conclusions on the expected actual pars plana vitrectomy (PPV) performance in retinal reattachment surgery.

## Material and Methods

Thanks to financial support from the German government and two foundations, the Frankfurt University Eye Clinic was able to start an extensive study focusing on the establishment of standard operation procedures (SOPs), success criteria in the field of retinal reattachment surgery and implementation of simulator-based practice.

The study was approved by the Ethical review committee University Frankfurt/M (IRB decision no. E 190/11, transaction no. 403/11), part of the study plan was the simulator-based warmup and performance test in advance of surgical procedures that is discussed in this article. The Ethical review committee also approved the written participant consent, which was provided by all participants.

This study was conducted in accordance with the Tenets of the Declaration of Helsinki. Patients’ records were pseudonymized and de-identified prior to statistical analysis.

In the preparation stage of the test, four participating surgeons performed several identical simulator courses within a two-week period. In the course of the paper, these surgeons are referred to as “user NN” with NN representing years of practice. The preparation stage was designed to make the participants familiar with the specific properties of the simulated environment.

During the study, the simulator software version 2.7.6 (release date: 24th of October 2011) was used. The simulator was equipped with a widefield viewing system and a vitreoretinal forceps handpiece.

In the surgery stage, we looked at PPVs for management of complex retinal detachment cases [21] performed by surgeons after a break from work of at least 60 hours.

After assigning a case to the best-suited surgeon for the surgery at hand, a starting point was determined with a simple 1:1 randomization scheme: either the surgeon went to the operating room immediately or he performed the simulator training courseware (duration: approximately 20 minutes) prior to going to the operating room. All surgical interventions were video-recorded and subsequently analyzed by two “blinded” observers (surgical vitreoretinal experience of more than 20 respectively more than 5 years). The more experienced observer graded the videos twice to allow a discussion of intraobserver consistency.

### Simulator courseware

The simulator training comprised an abstract bimanual task, two peelings (non-dominant and dominant hand) and a simulated retinal detachment surgery.

The following tasks were used:

- Bimanual Scissors Training Level 2 –Trains precise, simultaneous, asynchronuous instrument movements (aspirator, scissors) close to the retina.
- ILM Peeling Level 5 –Advanced task with a slightly stained, brittle membrane, i.e. frequent regrasping is necessary.
- ILM Peeling Level 5 –In this second ILM task with similar tissue properties, the peeling must be done with the non-dominant hand.
- Retinal Detachment Level 1 –Rhegmatogenous detachment with a horseshoe tear in the temporal periphery of a right eye, i.e. for some steps, a left-handed, nasal access is required.

All tasks were selected to match the nature of the real surgery–demanding vitreoretinal, bimanual PVR surgery. We specifically added an abstract task for bimanual skills training because, to our experience, the deliberate practice [22] of an isolated skill enables even experienced surgeons to raise their established performance level in this particular skill.

### Simulator scoring metrics

The simulator score is composed of numerous parameters. Each parameter has a specific point range that indicates its maximal influence. The total score is calculated by adding the score of all target parameters and subtracting the faults. The targets add up to 100 points whereas the sum of all faults can be higher. If so, the resulting score is cut off at zero points. Adding the fault scores instead of taking the average has been implemented in the simulator in analogy to the real situation: a surgical performance that is unsatisfying under multiple aspects adds up to an even worse situation. This is important in a training setup where predefined performance levels must be reached. For details see S1 Table.

In the realm of retinal detachment surgery, we identified the following primary targets: (1) amount of peeled ILM in macular area, (2) amount of reattached retina, and (3) amount of tractive tissue removed from the detached retina.The last parameter comprises the removal of enrolled edges of retinal tissue, of vitreous residuals, and of pigment and blood cells around retinal tears.

For the simulator these primary targets were scored according to S2 and S3 Tables.

### Scoring metrics of the video analysis

The scoring metrics for the video analysis were derived from the set of parameters measured by the simulator. Some parameters in the simulation (e.g. “fovea/optic disc/vessel hit by laser”) were combined into one parameter for the video analysis (e.g. “retinal injury due to laser energy”) or omitted due to minor surgical relevancy or inaccessibility (e.g. peeled ILM outside macular area, vitrector suction on retina).

**Fig 1:**
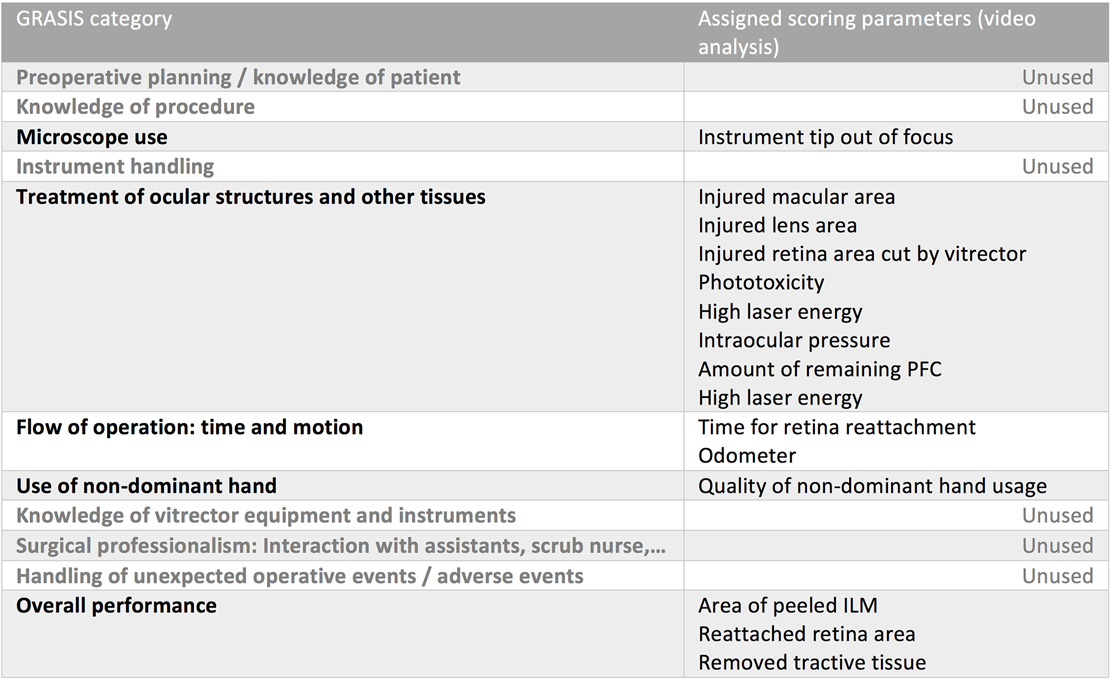
GRASIS categorization of the parameters as used by the video analysis.

GRASIS (Global Rating Assessment of Skills in Intraocular Surgery) is a validated scoring system for ophtalmic surgical competency [23]. As Fig 1 illustrates, our scoring metrics parameters can be categorized according to GRASIS. Note that there are differences: some GRASIS categories are not used at all (e.g. “Preoperative planning“, “Surgical professionalism“). They apply to a surgical novice rather than to a practicing surgeon. The three primary surgical targets were added to the GRASIS “Overall Performance”category, since GRASIS has no “targets”category.

The number of parameters assigned to each category varies considerably (from 7 parameters in the “Treatment of ocular structures”category to 1 parameter in “Microscope use”and “Use of non-dominant hand“). The total score is calculated by averaging over all parameters. Therefore the GRASIS categories do not contribute with equal weight. While this reflects the importance of a category in our setup quite well, it also represents a distortion of the GRASIS metrics. In order to support the validity of our approach, we performed an inter- and intragrader analysis of the video rating.

### Data pool and statistical method

The simple randomization resulted in the following distribution (total / with warmup / without warmup): user 02 (2 / 2 / 0), user 03 (6 / 2 / 4), user 07 (6 / 3 / 3), user 25 (7 / 2 / 5), i.e. 9 out of 21 cases were performed with simulator-based warmup whereas 12 cases were not preceeded by simulator trainingFor the examination of the warmup effect cases with warmup were compared to cases without warmup for each surgeon. For the 9 cases with warmup a detailed comparison of simulator and surgical performance was carried out.

Due to the special setup (N surgeons perform M interventions), we used a mixed-model approach for repeated measurements [24]. For illustrating the relation between performance on the simulator and in the operating room, we performed a linear regression after Pearson.For all statistical evaluations a p<0.05 was considered statistically significant. All the analyses were performed using BiAS V10.12 [25] for Windows and the R package V3.1–120 [26].

## Results

### Inter- and intragrader consistency

The grading of the recorded surgeries was performed by two independent vitreoretinal experts.

The Shrout-Fleiss intraclass correlation with a two-way mixed, single measure model resulted in a correlation coefficient of ICC (3,1) = 0.97 and a 95% confidence interval of 0.872…0.994.

In addition, an intra-individual regrading was performed. Here, the Bland-Altman intraclass correlation resulted in a correlation coefficient of ICC = 0.99 with a 95% confidence interval of 0.981…0.998.

### Effect of warmup training

The warmup training increases the final outcome in the operating room by 0.5 to 1 points in the video analysis. The statistical analysis with a mixed-model approach for repeated measurements showed a significant effect (p = 0.0302).

Warmed up or not, the standard deviation (SD) of the three less experienced surgeons (2/3/7 years of experience) is rather high with SD = 0.76 (n = 14) whereas the scores of the expert surgeon (25 years of experience) vary considerably less with SD = 0.21 (n = 7). An f-test showed (p = 0.004) that this difference between the less experienced surgeons and the expert is significant.

In the following it will be investigated if this surgical variability can be predicted by analyzing the simulator scores of the warmup training.

### Simulator-based prediction of operating room performance

**Fig 2:**
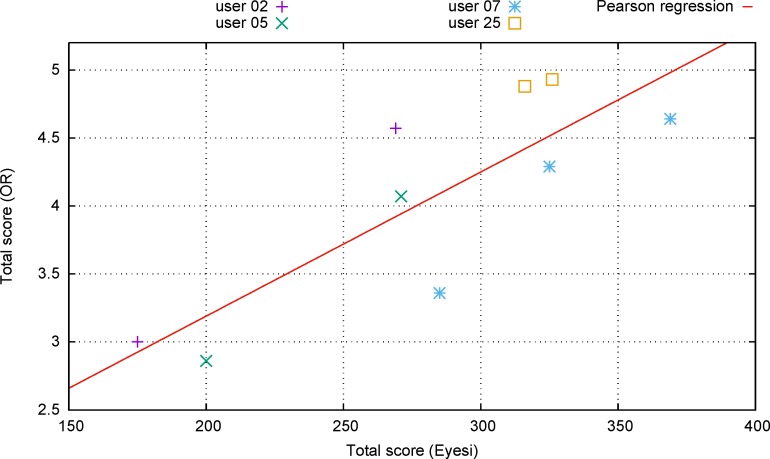
Comparison of the Eyesi score and the total score in the operating room. Individual surgeons are marked by different symbols. The experience of each surgeon in years is shown as part of his user name. The linear fit was determined by using a Pearson regression.

Fig 2 compares the scores achieved in the simulation and in the operating room. In general, the graph shows that performance varies from day to day for all surgeons, both on the simulator and in the operating room, and that these variations correlate.

In order to account for within-surgeon correlation we used a mixed model approach. The repeated measure test showed a significance of p = 0.000342 and a good model fit of R^2^ = 0.986.

For illustration, a linear regression after Pearson was performed and brought up a consistent result with p = 0.00603.

Table 1 shows the most prominent faults during simulated surgery; Listed are all penalties with more than 10 points off. Both the total amount of deduction (sum of all penalties) and the number of occurrences of a fault are shown in the table. The determinants of low scores on the simulator were lens damage, retinal bleeding and iatrogenic retinal holes.

**Table 1:** The most prominent “faults” during simulator training.

Scoring Parameter	Total Penalty	Number of Events
Injured lens area	-346	4
Macular spotted hemorrhages	-125	8
Injured (extramacular) retina area	-106	4
Retinal tear	-90	5
Injured macular area	-59	3
Vessel hit by laser	-50	5
Intact retina area cut by vitrector	-32	2
Amount of remaining PFC in the eye	-25	1
Extramacular spotted hemorrhages	-24	2
Phototoxicity	-16	1
Intraocular pressure too high or too low	-15	1

The table shows all events with a penalty of more than 10 points.

**Fig 3:**
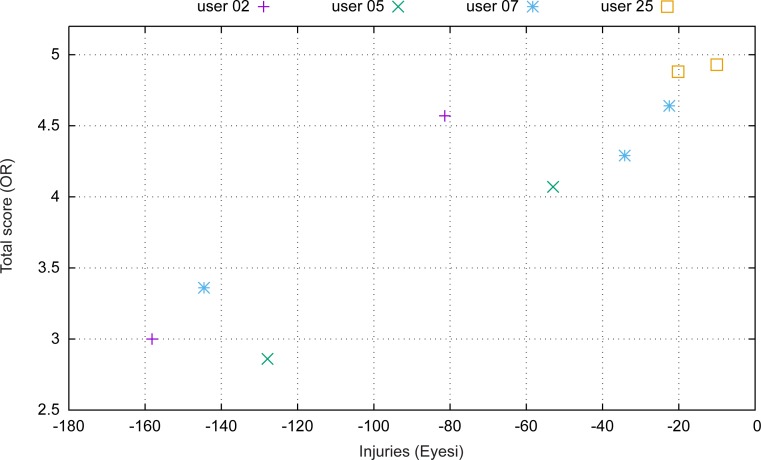
Comparison of injury score in simulation and total operating room score.

In Fig 3, the relation between injuries in the simulator and surgical performance is investigated. The figure compares for each user the total penalty for injuries with the performance in the real surgery. It is worth mentioning that a similar relation exists between the overall simulator score and the surgical performance as shown in Fig 2.

**Fig 4:**
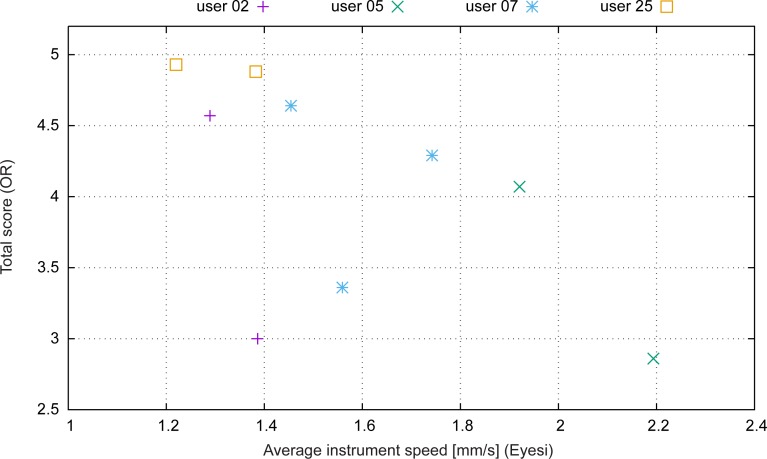
Average instrument speed (mm/s) in simulation in comparison to the performance in the operating room.

Fig 4 shows the operating room score in relation to the speed of the instrument movement in the simulator. The average instrument speed was determined by dividing the Eyesi odometry value (travelled distance of instrument tip) by the time taken. Apparently slower movements in Eyesi, i.e. a more precise, target-oriented approach, result in a better surgical performance.

The most important reasons for score deductions (deductions with more than 10 points off) in the simulator are listed in Table 2. As in Table 1, it is obvious that most deductions are based on tissue injuries–with one exception: the expert “user 25” did not only get less deductions, but, notably, they were limited to areas unrelated to tissue damage such as time and completion of targets.

**Table 2:** The most important reasons for deductions from the individual surgeon‘s score.

User	Task	Parameter	Deduction
user 05	Retinal Detachment	Injured lens area	-82.8
user 02	Retinal Detachment	Injured lens area	-63.3
user 07	Bimanual Scissors Training	Injured (extramacular) retina area	-47.8
user 07	Bimanual Scissors Training	Injured (extramacular) retina area	-32.9
user 07	Bimanual Scissors Training	Retinal tear	-30.0
user 05	Retinal Detachment	Stabilization of tears	-27.0
user 05	Retinal Detachment	Stabilization of tears	-23.0
user 05	Retinal Detachment	Removed tractive tissue	-20.0
user 05	ILM Peeling (non-dom.)	Macular spotted hemorrhages	-20.0
user 07	Bimanual Scissors Training	Completed objects	-20.0
user 02	ILM Peeling	Macular spotted hemorrhages	-20.0
user 02	ILM Peeling (non-dom.)	Macular spotted hemorrhages	-20.0
user 07	Bimanual Scissors Training	Injured macular area	-19.6
user 02	ILM Peeling (non-dom.)	Injured macular area	-18.3
user 07	Retinal Detachment	Stabilization of tears	-18.0
user 02	Retinal Detachment	Intact retina area cut by vitrector	-17.5
user 07	ILM Peeling (non-dom.)	Intraocular pressure too high or too low	-15.4
user 02	ILM Peeling (non-dom.)	Injured (extramacular) retina area	-15.2
user 05	Bimanual Scissors Training	Retinal tear	-15.0
user 02	Bimanual Scissors Training	Retinal tear	-15.0
user 07	Bimanual Scissors Training	Retinal tear	-15.0
user 07	Bimanual Scissors Training	Dissected objects	-14.3
user 02	Retinal Detachment	Intact retina area cut by vitrector	-14.2
user 02	ILM Peeling (non-dom.)	Spotted hemorrhages	-14.0
user 05	Retinal Detachment	Removed tractive tissue	-12.4
**user 25**	**ILM Peeling (non-dom.)**	**Peeled ILM outside macula area**	**-12.4**
**user 25**	**Retinal Detachment**	**Stabilization of tears**	**-12.0**
user 07	Retinal Detachment	Stabilization of tears	-12.0
**user 25**	**ILM Peeling**	**Peeled ILM outside macula area**	**-11.0**
user 02	ILM Peeling	Peeled ILM outside macula area	-11.0
user 02	Retinal Detachment	Stabilization of tears	-10.5
user 02	ILM Peeling	Peeled ILM removed from eye	-10.4
**user 25**	**ILM Peeling (non-dom.)**	**Peeled ILM inside macula area**	**-10.4**
user 02	ILM Peeling (non-dom.)	Injured (extramacular) retina area	-10.2
**user 25**	**Bimanual Scissors Training**	**Time**	**-10.1**
user 05	Bimanual Scissors Training	Dissected objects	-10.1

For the expert “user 25” (rows marked in boldface), fewer points are deducted and the losses are focused on target criteria, whereas all other users get the most deductions for injuries.

## Discussion

The aim of this study was to investigate whether and to what extent a warmup on the surgical simulator Eyesi is efficient in preparing the surgeon for an upcoming intervention. In addition it was investigated whether the performance parameters of the warmup training have predictive power regarding the outcome of the subsequent surgery.

VR to OR studies are still the exception because trials are difficult to carry out from an ethical standpoint. In our study design we chose the best surgeon available for a given maneuver. After making this choice, the surgeon was randomly assigned to either go straight to the operating room or to warm up at the simulator prior to the surgery.

Of all studies on Eyesi evaluating the role of training in either the anterior or the posterior eye segment, to our knowledge, there is none that investigates the warmup effect of simulator training.

Regarding the second part of the study, only Gill et al [20] used a similar setup and investigated the relationship between simulator performance and surgical performance. However they concentrated on beginners in cataract surgery whereas we focused on the advanced and expert vitreoretinal surgeon.

Despite the limited data set and the large variation of conditions (four surgeons, treating different pathologies, 21 samples), a statistically significant effect of warmup training on the surgical performance was found as well as a significant relation between performance on the simulator and in the operating room.

The average performance level of all surgeons was increased by the warmup training. Surprisingly, we found that even the most experienced surgeons benefit from warmup training.

However, the short training does not have an impact onto the variability of the performance level. We presume that a considerably longer training would be necessary to bring less experienced surgeons to a level of higher reliability that persists longer than the warmup effect that we observed: a 20 minutes warm up module cannot compensate for a lack of systematic training.

The performance values that were taken as the basis for the statistical evaluation were obtained by measuring numerous different parameters and either calculating a weighted sum (for the simulator score), or taking their average (for the video analysis of real surgery).

### Parameter analysis

The analysis of individual parameters or groups of parameters allows further insight into the training behavior and the relationship between simulation and real surgery. The ability to map an individual training detail to the corresponding surgical pattern is important for the design of individual simulator courses for training and warmup. In the following, we will discuss the relevance of our primary target parameters (ILM peeling, reattached retina, removed tractive tissue), of iatrogenic damages and, as an example of a parameter that can be measured much easier in simulation than in a video analysis, of the speed of instrument movements.

#### Injuries

Eyesi reacts extremely sensitively to undesirable injuries (Fig 3). This is intentional, since avoiding iatrogenic damage of all kinds was considered to positively affect the “over all” surgical performance. As we can see in this study, this is of utmost importance for those in the earlier stages of their surgical education, as they have a higher rate of iatrogenic failures compared to more experienced surgeons.

#### ILM peeling

ILM peeling seems to be a fairly standardized procedure. All surgeons peel around 44–50% ILM between the vessel arcades. However, there are significant differences in regard to the peeling quality and the percentage of injuries caused during the peeling. Some curiosities are worth to be mentioned: an increasing level of experience in vitreoretinal surgery might affect the surgeon’s decision to choose training elements they perceive as most needed (this was confirmed by the expert surgeon, who explained why he had not finished the ILM peel in Eyesi: “I shortened the Eyesi training module because I know that once the beginning of a peel works well the rest will flow smoothly anyway”). The expert tends to overrule the simulator‘s operation procedure by making use of individually composed training steps. While this might call into question the measurability of expert performance, at the same time it constitutes a training value in itself, as the artificial environment makes it possible to isolate relevant training steps, thus increasing learning curves and the efficiency of the training (deliberate practice).

#### Reattached retina

Both in the simulator and in the real surgery, intraoperative reattachment of the retina could be achieved in 100% of the performed tasks. Therefore this parameter is not suitable for a discriminative analysis of warmup effects.

#### Removed tractive tissue

For a standardized treatment of rhegmatogenous retinal detachment with PVR activity, the removal of tissue around retinal tears has been identified as one important surgical step in our hospital. Therefore it was also integrated into the simulated surgery and is the third target criterium in our setup. Working on a tear in the periphery with the appropriate configuration of the surgical machine is a delicate part of the surgery. We see a relationship between the performance at the simulator and in the operating room–with one exception: user 05, who performed well in real surgery, but not on the same level in the simulator.

#### Instrument movements

By comparing the score in the operating room and the instrument speed while managing a retinal detachment (Fig 4), we noticed that more expertise correlates with slower movements and less “action” in the vitreous cavity. This seems to be a bit of a surprise first–but is easily explained by the fact that during the vitreoretinal education, every surgeon is told over and over that the slower and more precise she or he moves the instruments, the more efficiently and ultimately faster the procedure will be finished. Eyesi records all movements, classifies them as necessary or unnecessary and scores them with the “odometry” measurement.

Fig 4 shows two exceptional situations (at 1.4/3 and 1.58/3.4): two “non-expert-surgeons” faced two different situations which Eyesi had not prepared them for: the first one was a perfluorocarbon-to-air-to-silicon-oil exchange which was not available in the simulation at that time. The second situation was an inappropriate set of instruments (highly myopic eye, instrument too short) which did not allow an adequate peeling of the ILM as planned, resulting in repeated time-consuming and ultimately unsuccessful approaches (readjusting the microscope, changing dominant and non-dominant hand, etc.).

While the perfluorocarbon-to-air-to-silicon-oil exchange has been added meanwhile, the simulator is not yet able to provide situations with myopic eyes where the right strategies to shorten the actual length of the eye can be trained.

### Strength and limitations of this paper

This paper presents true VR-to-OR data about training of several vitreoretinal surgeons with different surgical expertise. This could not be found in literature up to now. Due to ethics considerations (“choose the most suitable surgeon first”), we did not know beforehand which surgeon had to perform how many interventions. In combination with the “weekend situation”, this resulted in a limited number of cases (n = 21). The simple randomization scheme that we used led to one surgeon performing real surgery after warmup only.

Despite these limitations, our mixed model approach showed significant results. For future studies with a similar setup, we suggest to replace the simple randomization scheme by a block randomization with fixed A/B sequences (example: 5 surgeons with 3 A/B sequences, resulting in 30 cases). Although this would prolongate the data acquisition, it would guarantee an equal distribution that ultimately results in a higher statistical power. Note that this design resembles an N-of-1 trial series, especially with regard to basic design considerations, such as randomization and carryover effects [27].

Since this kind of study has not been published before, it is difficult to find a rating system in literature we can refer to: There is limited possibility of comparison between the global rating assessment GRASIS which focusses on surgeons in their early stage of education and the assessment adjusted for this study which looks atthe performance of surgeons in their early and later stages of education including experts. It seems to be an advantage that in this study different skill levels were covered and less experienced surgeons as well as advanced surgeons and experts were included into the data pool.Further questions have to be addressed and answered in consecutive studies. We chose a break of 60 hours to reflect a weekend situation. How do longer or shorter breaks affect the observed drop in performance? Are surgeons in average more (or less) confident after they used the simulator? Does the level of confidence correlate with the measurable skill performance? Could the observed relation between simulator and surgical performance be used to define a threshold for entering the operating room? Should a specific courseware be designed for experts which allow them to choose between different roads and adjust the training to the individual needs under variable conditions, such as different time intervals between surgical interventions, anticipated complications, different stress levels etc.

And finally: what clinically relevant simulations must be added to increase the efficiency of a simulator-based warmup and performance measurement?

## Supporting Information

S1 Table Scoring parameters that all simulated tasks have in common. Since the targets are task-specific, the table only shows faults and the neutral parameter “Odometer” that measures the travelled distance of the instruments tips. This parameter is needed for an analysis of the surgeons’ instrument speed.
(PDF)

S2 Table Specific scoring parameters for simulated ILM peeling. The primary target is marked with a star (*).
(PDF)

S3 Table Specific scoring parameters for simulated retinal detachment surgery. Target parameters are indicated by a positive, faults by a negative point range. The primary targets are marked with a star (*).
(PDF)
