# Supplementary material for: Clinical Efficacy of Simulated Vitreoretinal Surgery to Prepare Surgeons for the Upcoming Intervention in the Operating Room
Source: PLoS One. 2016 Mar 10;11(3):e0150690. doi: 10.1371/journal.pone.0150690 (PMC4786212; doi:10.1371/journal.pone.0150690)
Supplement: S1 Table — Since the targets are task-specific, the table only shows faults and the neutral parameter “Odometer” that measures the travelled distance of the instruments tips. This parameter is needed for an analysis of the surgeons’ instrument speed. (PDF) [file pone.0150690.s001.pdf]

S1 Table. Scoring parameters that all simulated tasks have in common. Since the targets are task-specific, the table only shows faults and the neutral parameter “Odometer” that measures the travelled distance of the instruments tips. This parameter is needed for an analysis of the surgeons’ instrument speed.

**Scoring parameter**	**Point range**
Injured macular area	-100
Macular spotted hemorrhages	-100
Injured extramacular area	-100
Extramacular spotted hemorrhages	-100
Retinal tear	-100
Phototoxicity	-100
Injured lens area	-100
Intraocular pressure too high or too low	-100
Vitrector suction on retina	-50
Time	-20
Instrument tip out of focus	-20
Open forceps insertion/removal	-20
Odometer	0
