# Supplementary material for: Clinical Efficacy of Simulated Vitreoretinal Surgery to Prepare Surgeons for the Upcoming Intervention in the Operating Room
Source: PLoS One. 2016 Mar 10;11(3):e0150690. doi: 10.1371/journal.pone.0150690 (PMC4786212; doi:10.1371/journal.pone.0150690)
Supplement: S2 Table — The primary target is marked with a star (*). (PDF) [file pone.0150690.s002.pdf]

S2 Table. Specific scoring parameters for simulated ILM peeling. The primary target is marked with a star (*).

Scoring parameter	Point range
Peeled ILM in macular area*	+50
Peeled ILM outside macular area	+20
Peeled ILM removed from eye	+30
