# Supplementary material for: Clinical Efficacy of Simulated Vitreoretinal Surgery to Prepare Surgeons for the Upcoming Intervention in the Operating Room
Source: PLoS One. 2016 Mar 10;11(3):e0150690. doi: 10.1371/journal.pone.0150690 (PMC4786212; doi:10.1371/journal.pone.0150690)
Supplement: S3 Table — Target parameters are indicated by a positive, faults by a negative point range. The primary targets are marked with a star (*). (PDF) [file pone.0150690.s003.pdf]

S3 Table. Specific scoring parameters for simulated retinal detachment surgery. Target parameters are indicated by a positive, faults by a negative point range. The primary targets are marked with a star (*).

**Scoring parameter**	**Point range**
Reattached retinal area*	+50
Removed tractive tissue*	+20
Stabilization of retinal tears (by endolaser)	+30
Remaining PFC in the eye	-100
Instrument near macula	-20
Misplaced laser patch	-100
Fovea hit by laser	-100
Optic disc hit by laser	-100
Vessel hit by laser	-20
High laser energy on retina	-10
Retinal injury due to laser energy	-100
